# Integrated Analysis to Evaluate the Prognostic Value of Signature mRNAs in Glioblastoma Multiforme

**DOI:** 10.3389/fgene.2020.00253

**Published:** 2020-03-31

**Authors:** Ji’an Yang, Long Wang, Zhou Xu, Liquan Wu, Baohui Liu, Junmin Wang, Daofeng Tian, Xiaoxing Xiong, Qianxue Chen

**Affiliations:** Department of Neurosurgery, Renmin Hospital of Wuhan University, Wuhan, China

**Keywords:** glioblastoma, GEO, TCGA, WGCNA, prognosis biomarkers

## Abstract

**Background:**

Gliomas are the most common intracranial tumors and are classified as I–IV. Among them, glioblastoma multiforme (GBM) is the most common invasive glioma with a poor prognosis. New molecular biomarkers that can predict clinical outcomes in GBM patients must be identified, which will help comprehend their pathogenesis and supply personalized treatment. Our research revealed four powerful survival indicators in GBM by reanalyzing microarray data and genetic sequencing data in public databases. Moreover, it unraveled new potential therapeutic targets which could help improve the survival time and quality of life of GBM patients.

**Materials and Methods:**

To identify prognostic signatures in GBMs, we analyzed the gene profiling data of GBM and standard brain samples from the Gene Expression Omnibus, including four datasets and RNA sequencing data from The Cancer Genome Atlas (TCGA) containing 152 glioblastoma tissues. We performed the differential analysis, Gene Ontology (GO) and Kyoto Encyclopedia of Genes and Genomes (KEGG) pathway analysis, weighted gene co-expression network analysis (WGCNA) and Cox regression analysis.

**Results:**

After differential analysis in GSE12657, GSE15824, GSE42656 and GSE50161, overlapping differentially expressed genes were identified. We identified 110 up-regulated DEGs and 75 down-regulated DEGs in the GBM samples. Significantly enriched subclasses of the GO classification of these genes included mitotic sister chromatid separation, mitotic nuclear division and so on. In KEGG pathway analysis, the most abundant terms were ECM-receptor interaction and protein digestion and absorption. WGCNA analysis was performed on these 185 DEGs in 152 glioblastoma samples obtained from TCGA, and gene co-expression networks were constructed. We then performed a multivariate Cox analysis and established a Cox proportional hazards regression model using the top 20 genes significantly correlated with survival time. We identified a four-protein prognostic signature that could divide patients into high-risk and low-risk groups. Increased expression of SLC12A5, CCL2, IGFBP2, and PDPN was associated with increased risk scores. Finally, the K-M curves confirmed that these genes could be used as independent predictors of survival in patients with glioblastoma.

**Conclusion:**

Our analytical study identified a set of potential biomarkers that could predict survival and may contribute to successful treatment of GBM patients.

## Introduction

Gliomas are the most common intracranial tumors and are classified as grades I–IV according to World Health Organization (WHO) Classification of Tumors of the Central Nervous System (CNS). Among them, glioblastoma multiforme (GBM) is the most common primary brain tumor in adults with a poor prognosis (Reni et al., 2017). Patients with glioblastoma multiforme usually survive for less than 15 months after diagnosis and treatment. Therefore, it is crucial to develop appropriate and effective biomarkers to predict the prognosis of patients with glioblastoma. Various tumor related biomarkers have been found in glioblastoma, including epidermal growth factor receptor (EGFR), mutant form of the EGFR (EGFRvIII), vascular endothelial growth factor (VEGF), p53 and Phosphate and tensin homolog deleted on chromosome 10 (PTEN), Retinoblastoma (RB1) and Isocitrate dehydrogenase (IDH) (Appin and Brat, 2015). Some of these markers can predict therapeutic effect and clinical prognosis (Garrett-Bakelman and Melnick, 2013; Network, 2013; Westphal and Lamszus, 2015). Methylation status of the promoter of O-6-methylguanine-DNA methyltransferase (MGMT) is related to the sensitivity of temozolamide therapy and the prognosis of patients (Hegi et al., 2005; Wang et al., 2018). Loss of heterozygosity (LOH) of 1p/19q is another prognostic indicator, representing a better prognosis (Wiestler et al., 2014; Zhao et al., 2014). However, these markers can only be applied to specific parts of glioblastoma patients, and their proportion is not high. It is still necessary to identify novel molecular biomarkers that can predict the clinical outcome of GBM patients, which could help comprehend their pathogenesis and supply personalized treatment.

**FIGURE 1:**
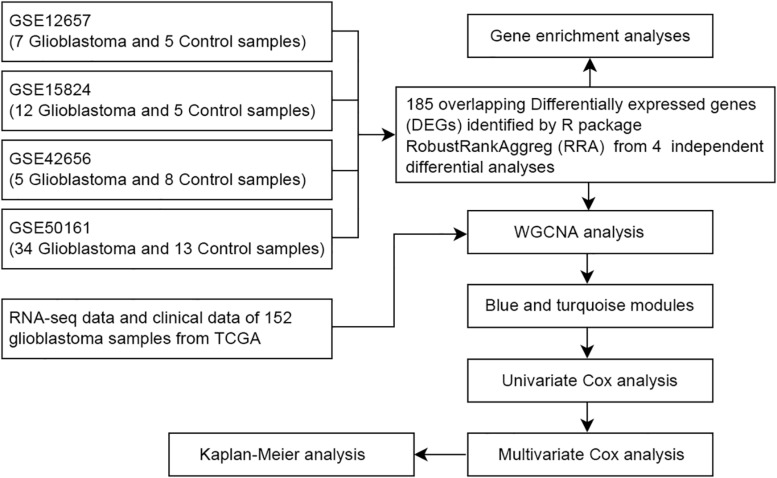
Flow chart of data collection and analysis.

With the rapid development of sequencing technology and bioinformatics, they have provided new ideas for the study of clinical problems and related pathological mechanisms of various cancers. The Gene Expression Omnibus (GEO), The Cancer Genome Atlas (TCGA) and other public databases are broadly integrated collections of microarray data and gene sequencing data, enabling investigators to perform systematic analysis, which can help improve the diagnostic methods and survival prognosis of cancer patients. Considering different detection methods used by different technological platforms, as shown in Figure 1, various data processing and analysis methods are being explored. In this study, the RobustRankAggreg (RRA) (Kolde et al., 2012) method was used to combine the results of several separate studies to improve statistical power. Meanwhile, weighted gene co-expression network analysis (WGCNA) (Fuller et al., 2007; Langfelder and Horvath, 2008) was adopted to construct free-scale gene co-expression networks to identify core genes associated with clinical outcomes. These core genes may have important clinical significance and can be used as diagnostic and prognostic biomarkers or therapeutic targets.

## Materials and Methods

### Microarray Data

Gene profiling data of GBM and normal brain samples were downloaded from the GEO^1^, a public functional genomics data repository. Four datasets were selected for bioinformatics analysis, including GSE12657 (GPL8300, Affymetrix Human Genome U95 Version 2 Array),GSE50161 (GPL570, Affymetrix Human Genome U133 Plus 2.0 Array) (Griesinger et al., 2013), GSE42656 (GPL6947, Illumina HumanHT-12 V3.0 expression chip) (Henriquez et al., 2013) and GSE15824 (GPL570, Affymetrix Human Genome U133 Plus 2.0 Array) (Grzmil et al., 2011). All raw data were downloaded from the GEO database.

### Microarray Data Normalization and Probe Annotation

The microarray data were quantile normalized using the “limma” package (Ritchie et al., 2015). After the data were normalized, the probe data in the original format were mapped to the gene symbols based on the annotation information. If multiple probes correspond to a gene, the average expression value of these probes was calculated as the expression of the gene (Xu et al., 2018). For probes with missing values, the “impute” package^2^ was used to fill in missing values.

### Download and Pre-processing of RNA-seq Data From TCGA

RNA sequencing data of human glioblastoma samples were available from the TCGA data portal^3^, which contained 152 glioblastoma tissues. These data were then constructed into a matrix of RNA sequences, where gene symbols were rows and patient barcodes were column names. The clinical metadata of 152 samples were also downloaded and filtered for useful information.

### Differential Analysis

Difference analysis was performed on four GEO datasets using the R package “limma” (Ritchie et al., 2015). In order to determine the best ranking results of the differential genes, a new robust rank aggregation method was used, which was implemented as the R package “RobustRankAggreg” (RRA)^4^ (Kolde et al., 2012).

### GO and KEGG Enrichment Analysis

The enrichment analysis of the KEGG pathway and Gene Ontology terms were performed through the R package “clusterProfiler”^5^ (Yu et al., 2012; Yu et al., 2015). Enriched ontological terms and pathways (*P* < 0.05) were visualized as histograms.

### Weighted Gene Co-expression Network Analysis

The R software package “WGCNA” was used for weighted gene co-expression network analysis (Langfelder and Horvath, 2008). It is an algorithm for constructing co-expression networks, defined by the similarity of gene co-expression. First, we calculated the Pearson correlation between each pair of differential genes and obtained a similarity matrix (sij). Second, the similarity matrix was converted into an adjacency matrix. The topological matrix was created using topological overlap measure (TOM) (Yip and Horvath, 2007). Finally, we chose the Dynamic hybrid cut method to identify co-expression gene modules (Langfelder et al., 2008). Details on the algorithm were available on request.

### Cox Regression Analysis

To validate the significance of the prognostic risk genes screened above, we used univariate Cox proportional hazards regression to assess the effect of expression of these genes on survival time in GBM patients. Limited to the strength of computer calculation, we used the top 20 genes significantly related to survival time to perform the multivariate Cox analysis. Then, statistically significant genes were used to construct a multivariate cox regression model. The above analysis had used the R package “survival”^6^ (Therneau and Grambsch, 2000). The R package “survivalROC”^7^ was used to perform the receiver operating characteristic curve (ROC) to evaluate the accuracy of the model (Heagerty et al., 2000).

### Statistical Analysis

All statistical tests and charts were performed using RStudio. *P* < 0.05 was considered statistically significant. These graphics were then integrated and displayed using Photoshop.

## Results

### Screening for Differentially Expressed Genes (DEGs)

**FIGURE 2:**
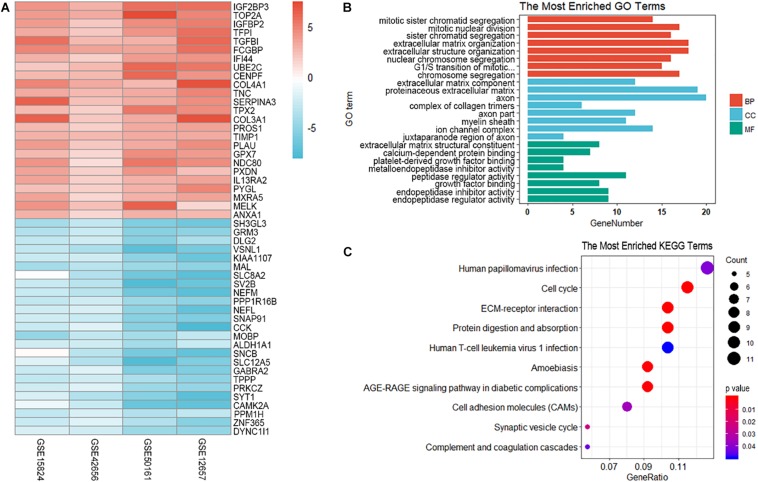
Visualization and enrichment analysis of differentially expressed genes. **(A)** The heatmap of the top 50 DEGs. **(B)** GO enrichment analysis of differentially expressed genes. BP, biological process; CC, cellular component; MF, molecular function; **(C)** KEGG enrichment analysis of differentially expressed genes. The size of the dot represents the count number of genes in one KEGG term.

The differential analysis in GSE12657, GSE50161, GSE42656, and GSE15824 was performed by “limma” algorithm. Subsequently, 185 overlapping differentially expressed genes were identified by “RobustRankAggreg,” of which 110 were up-regulated and 75 were down-regulated in GBM samples. The top 50 DEGs were visualized as heatmap (Figure 2A).

### GO and KEGG Enrichment Analysis of DEGs

To explore the biological relevance of DEGs, Gene Ontology (Ashburner et al., 2000) and KEGG (Ogata et al., 2000) pathway enrichment analyses were performed. GO and KEGG analysis predicted that these genes were involved in several important physiological processes. These genes were significantly enriched in the following subclasses of GO classification:mitotic sister chromatid segregation (GO: 0000070 *P* = 2.67E-10), mitotic nuclear division (GO: 0140014 1.28E – 09), sister chromatid segregation (GO: 0000819 *P* = 2.44E – 09), extracellular matrix component (GO: 0044420 *P* = 2.69E – 09),proteinaceous extracellular matrix (GO: 0005578 *P* = 4.39E – 09) and extracellular matrix structural constituent (GO: 0005201 *P* = 1.57E – 06). The KEGG pathway analysis showed that the most enriched terms were ECM-receptor interaction (hsa04512 *P* = 4.18E – 07), protein digestion and absorption (hsa04974, *P* = 9.33E – 07) (Figures 2B,C).

### Co-expression Network Construction and Visualization

**FIGURE 3:**
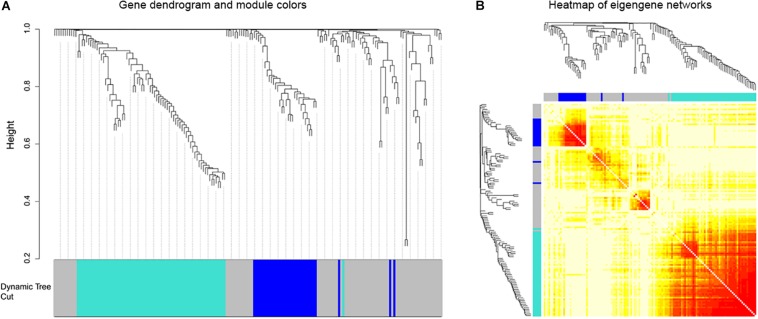
Weighted gene co-expression network of glioblastoma. **(A)** Gene dendrograms obtained by average linkage hierarchical clustering of 185 genes based on consensus Topological Overlap with the corresponding module colors indicated by the color row. **(B)** The eigengene networks were shown as heatmap. The deeper the color expressed a high adjacency.

**FIGURE 4:**
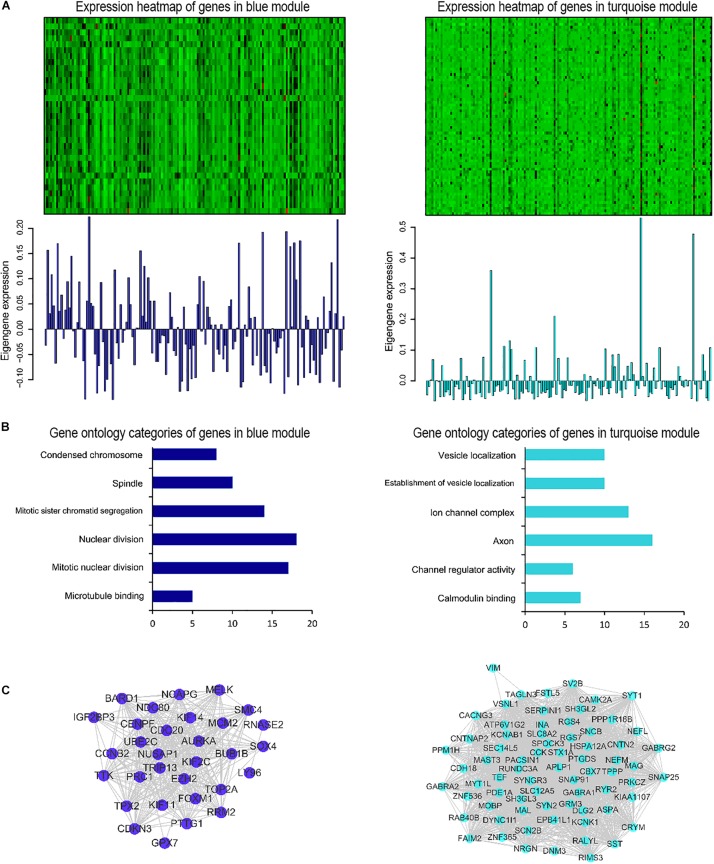
Gene co-expression modules associated with glioblastoma. **(A)** Heatmap of genes belonging to the co-expression module. Corresponding module eigengene values (y-axis) across samples (x-axis). **(B)** Relevant gene ontology categories of enriched genes in the blue and turquoise modules. **(C)** Visualization of the gene co-expression network of the blue and turquoise modules.

Afterward, the WGCNA analysis was performed to construct gene co-expression networks. We analyzed the 185 DEGs identified above in the data of 152 glioblastoma samples from TCGA and divided the 185 genes into three modules (Figure 3). The blue and turquoise co-expressed modules were identified to further analysis (Figure 4A). In order to explore whether different modules have different biological functions, enrichment analysis was also performed on the modules. It was found that the biological processes of the blue module mainly focused on cell proliferation and division. However, the turquoise module focused on signal molecule delivery (Figure 4B). Whereafter, the co-expression networks of the modules were exported into Cytoscape and visualized (Shannon et al., 2003). The nodes were defined as individual genes in the networks, and the edges were defined as the interactions between genes (Figure 4C).

### Construction of the Cox Proportional Hazards Regression Model Based on Hub Genes and Kaplan–Meier Analysis

**FIGURE 5:**
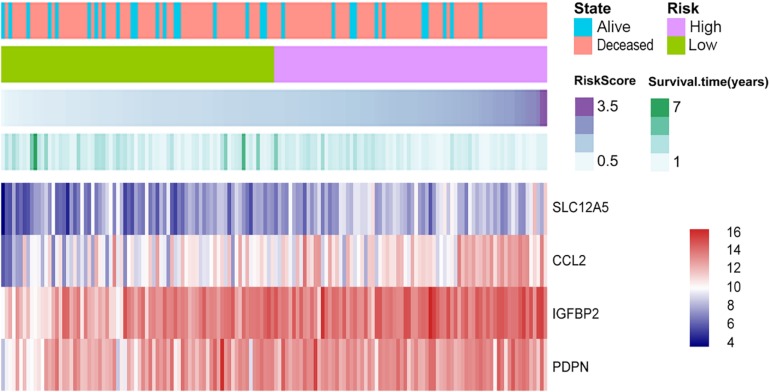
Cox proportional hazards regression model. Purple depths of the third column reveal the risk score of the low-risk and high-risk groups. Green depths of the fourth column display the survival status and time of 152 glioblastomas. The lowest column shows the heatmap of the model genes.

**FIGURE 6:**
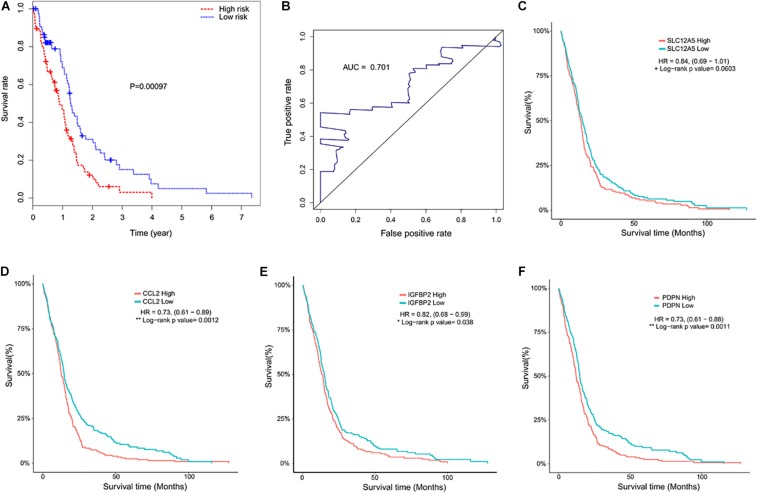
Kaplan–Meier curves and receiver operating characteristic (ROC). **(A)** Kaplan–Meier curve showed that the mortality in the high-risk group was higher than that in the low risk group (*P* < 0.001). **(B)** Time-dependent ROC curve indicated a higher predictive value. The area under the ROC curve (AUC) was 0.701. **(C–F)** Kaplan–Meier curves of the four predictive indicators.

The selected DEGs were further used to perform univariate Cox analysis. We then performed a multivariate Cox analysis using the top 20 genes significantly correlated with survival time, and constructed a Cox proportional hazards regression model from 152 patients with glioblastoma. Based on the above model, the following formula was used to calculate the risk score for predicting survival time: risk score = (0.2239^∗^expression level of CCL2) + (0.3375^∗^expression level of IGFBP2) + (0.1516^∗^expression level of PDPN) + (0.2276^∗^expression level of SLC12A5) (Figure 5). According to the median risk score, 152 patients were divided into high-risk (*N* = 76) and low-risk (*N* = 76) groups. The 5-year survival rate in the high-risk group was significantly lower than low-risk group. Increased expression of SLC12A5, CCL2, IGFBP2, and PDPN was associated with increased risk scores (Figure 6A). The area under the ROC curve was 0.701 (Figure 6B), indicating the high predictive value. Meanwhile, K-M curves confirmed that these three genes (CCL2, IGFBP2, and PDPN) could be used as independent predictors of survival in patients with glioblastoma (Figures 6C–F).

## Discussion

High-throughput microarray technology provides insights into pathogenesis, molecular heterogeneity and treatment response. The biological conclusions are inconsistent due to differences in detection platforms and laboratory protocols and noisy microarray data. To overcome these limitations, it is considerable to analyze these data set separately and then summarize different lists of results. In our research, we identified 185 DEGs for GBM derived from independent profiling datasets by applying “limma” algorithm and “RRA” method. This method using a probabilistic model probabilistic model makes the algorithm parameter free and robust to outliers, noise and errors, and facilitates the calculation of significance probabilities for all the elements in the final ranking. This strategy has been widely applied to identify disease-related genes (Kolde et al., 2012; Xiao, 2020; Xiong et al., 2018).

Subsequently, the WGCNA analysis was performed on RNA-seq data obtained from TCGA on those 185 DEGs to identify two co-expressed modules (blue and turquoise). WGCNA is a recently developed method to construct a weighted gene co-expression network and a new analytic approach to move beyond single-gene comparisons (Giulietti et al., 2018). The WGCNA algorithm has been used to identify disease-related genes, biological pathways and therapeutic targets for diseases such as familial combined hyperlipidemia, Osteoporosis, Autistic, and Alzheimer disease (Goh et al., 2007; He et al., 2011; Tang et al., 2017). It also has been used in neuroscience and oncology. Michael C Oldham performed the WGCNA in normal human brains to identify co-expressed gene modules that reflected the underlying cellular composition of brain tissue and system-level molecules related to neuroanatomy (Oldham et al., 2006). The large number of tumor RNA-seq data and other high-throughput data resources such as TCGA provide a broad opportunity for the application of WGCNA in cancer research. To date, there have been similar studies on gliomas. Zhou and colleagues revisited the gene expression profile data downloaded from GEO to identify novel genes associated with pediatric pilocytic astrocytoma using the WGCNA analysis. They identified nine network modules associated with pilocytic astrocytomas. The further functional analysis revealed that these genes were involved in the regulation of cell differentiation (Zhou and Man, 2016). S. Horvath used WGCNA to identify several gene co-expression modules and revealed abnormal spindle-like microcephaly-associated protein (ASPM) that might function as a potential molecular target in glioblastoma (Horvath et al., 2006). In addition, Upton A and his colleagues used the WGCNA algorithm and further identified 92 genes that were associated with different evolutionary stages of glioblastoma (Upton and Arvanitis, 2014). In our research, the biological processes of the blue module mainly focused on cell proliferation and division. While, the turquoise module focused on signal molecule delivery. These results help to understand the occurrence and development of glioblastoma to some extent, and further research is needed.

Cox proportional hazards regression has been widely used to examine the prognostic value of candidate predictors in human diseases (Degnim et al., 2018; Liu et al., 2018). Aoki K used the Cox proportional hazards regression model to study the effects of genetic variation and clinicopathological factors on the survival of diffuse low-grade gliomas (LGGs). The authors reported subtype-specific genetic alterations could stratify patients with different LGG subtypes (Aoki et al., 2018). By constructing the Cox proportional hazards regression model, we selected an optimal four-gene model (SLC12A5 + CCL2 + IGFBP2 + PDPN) for prognosis prediction. Among the genes in this model, solute carrier family 12, member 5 (SLC12A5) was considered as a neuron marker, but it has not been reported in glioma-related studies. Chemokine ligand 2 (CCL2) is one of several cytokine genes and could be secreted by astrocytoma cells and myeloid cells. Importantly, CCL2 then recruits regulatory T cells (Tregs) and myeloid-derived suppressor cells (MDSCs) through CCR4 and CCR2 as significant contributors to the potently immunosuppressive glioma microenvironment (Carrillo-de et al., 2012; Braganhol et al., 2015; Chang et al., 2016; Lu et al., 2017). Overexpression of Insulin-like growth factor binding protein 2 (IGFBP2) has been reported to be involved in the progression of many types of cancer. In gliomas, IGFBP2 is considered to be an oncogene that causes glioma progression through integrin/ILK/NF-kB pathway (Phillips et al., 2016). According to reports, Podoplanin (PDPN) was a novel candidate gene that might play an essential role in glioblastoma pathogenesis and response to treatment (Sailer et al., 2013; Krishnan et al., 2018). However, these genes and the related signaling pathways and mechanisms involved are still not clear enough.

Our research has some limitations. First, in order to reduce intensity of computer operation, we used the top 20 genes significantly related to survival time to perform the multivariate Cox analysis. But constructing a model with more genes might get more meaningful results. Second, due to the lack of survival data in the GEO datasets, we did not validate the prognostic value of the four-gene model. Third, the expression levels of corresponding proteins have not been verified in tissue samples. Finally, we used the “RRA” method to identify DEGs, and in this process, the tumor heterogeneity might be ignored. We might lose some key genes and pathways in the development of gliomas in the integration analysis. In summary, in this study, we tried to apply a new procedure to screen out some new biomarkers that can help the diagnosis and treatment of glioblastoma. Although the methods are not new, combining them with new process may bring new perspectives. We identified a four-gene (SLC12A5 + CCL2 + IGFBP2 + PDPN) Cox proportional hazards regression model for prognosis prediction. Although the specific mechanism remains to be studied, these genes could be considered as risk factors for GBM patients and novel therapeutic targets.

## Data Availability Statement

Microarray data were retrieved from the GEO data repository (http://www.ncbi.nlm.nih.gov/geo/) with the accession numbers: GSE12657, GSE50161, GSE42656, and GSE15824. The RNA sequencing data of human glioblastoma samples were obtained from the TCGA data portal (https://portal.gdc.cancer.gov).

## Author Contributions

JY contributed to the publication search, data extraction, draft writing, and conception and design. LW, ZX, LqW, JW, DT, XX, and QC contributed to the quality assessment, conception and design, and editing. BL contributed to the statistical analysis.

## Conflict of Interest

The authors declare that the research was conducted in the absence of any commercial or financial relationships that could be construed as a potential conflict of interest.

## Footnotes

1 http://www.ncbi.nlm.nih.gov/geo

2 http://bioconductor.org/packages/release/bioc/html/impute.html

3 https://cancergenome.nih.gov/

4 https://CRAN.R-project.org/package=RobustRankAggreg

5 https://github.com/YuLab-SMU/clusterProfiler

6 https://CRAN.R-project.org/package=survival

7 https://CRAN.R-project.org/package=survivalROC
